# Alcohol induces cell proliferation via hypermethylation of *ADHFE1* in colorectal cancer cells

**DOI:** 10.1186/1471-2407-14-377

**Published:** 2014-05-28

**Authors:** Ji Wook Moon, Soo Kyung Lee, Yong Woo Lee, Jung Ok Lee, Nami Kim, Hye Jeong Lee, Jung Seon Seo, Jin Kim, Hyeon Soo Kim, Sun-Hwa Park

**Affiliations:** 1 Institute of Human Genetics, Department of Anatomy, Korea University College of Medicine, 126-1, Anam-dong 5-ga, Seongbuk-gu, Seoul 136-705, Republic of Korea; 2 Department of General Surgery, Korea University Medical Center, 126-1, Anam-dong 5-ga, Seongbuk-gu, Seoul 136-705, Republic of Korea

**Keywords:** *ADHFE1*, Colorectal cancer, Hypermethylation, Alcohol, Quantitative methylation-specific polymerase chain reaction

## Abstract

**Background:**

The hypermethylation of Alcohol dehydrogenase iron containing 1 (*ADHFE1*) was recently reported to be associated with colorectal cancer (CRC) differentiation. However, the effect of alcohol on *ADHFE1* hypermethylation in CRC is still unclear.

**Methods:**

The methylation status and expression levels of *ADHFE1* were investigated in primary tumor tissues and adjacent normal tissues of 73 patients with CRC, one normal colon cell line, and 4 CRC cell lines (HT-29, SW480, DLD-1, and LoVo) by quantitative methylation-specific polymerase chain reaction (QMSP) and real-time reverse transcription polymerase chain reaction (real time PCR), respectively. The effect of alcohol on the methylation status of *ADHFE1* was analyzed in HT-29, SW480, DLD-1, and CCD18Co cells using QMSP, real-time PCR, immunoblot, and cell proliferation assay.

**Results:**

*ADHFE1* was hypermethylated in 69 of 73 CRC tissues (95%) compared to adjacent normal tissues (*p* < 0.05). The mRNA expression of *ADHFE1* was significantly reduced in CRC compared to adjacent normal tissues (*p <* 0.05) and its expression was decreased in the alcohol consumption group (*p <* 0.05). *ADHFE1* was hypermethylated and its expression was decreased in 4 CRC cell lines compared with normal colon cell line. Alcohol induced hypermethylation of *ADHFE1*, decreased its expression, and stimulated cell proliferation of HT-29, SW480, and DLD-1cells.

**Conclusion:**

These results demonstrate that the promoter hypermethylation of *ADHFE1* is frequently present in CRC and alcohol induces methylation-mediated down expression of *ADHFE1* and proliferation of CRC cells.

## Background

Aberrant DNA methylation leads to the suppression of tumor suppressor gene expression as an epigenetic events [1]. In mammals, DNA methylation occurs only at CpG dinucleotide pairs, in which a 5′ cytosine residue is situated adjacent to a guanine residue. Hypermethylation of CpG-rich or intermediate promoters has been shown to inactivate downstream gene expression, and promoter hypermethylation of tumor suppressor genes is frequently observed in many human malignancies, and may contribute to disease pathogenesis [2-5]. Colorectal cancer (CRC) is one of the most common cancers in Korea, and its incidence has been steadily increasing. Epigenetic alterations that are commonly found in CRCs include DNA methylation of tumor suppressor genes [6,7] and histone deacetylation [8].

Alcohol overconsumption is a well-known risk factor for the development as well as progression of various types of cancers, including CRC [9]. When alcohol is consumed, it is metabolized by alcohol dehydrogenases (ADH) and cytochrome P450 subenzyme 2E1 (CYP2E1), which catalyze the oxidation of alcohol to acetaldehyde [10]. Alcohol dehydrogenases (*ADH*) are a well-defined group of enzymes involved in general detoxification of alcohols and are associated with several human cancers, including CRC [11]. Alcohol metabolism increases reactive oxygen species (ROS) generation, resulting in oxidative stress [12], and accumulation of ROS enhances arsenic-induced tumor angiogenesis in CRC cells via the HIF-1α pathway [13]. Alcohol promotes cancer progression by inducing gene expression of epithelial-mesenchymal transition (EMT) genes, such as the transcription factor Snail, by increasing epidermal growth factor receptor (EGFR) transactivation and activating matrix metalloproteinases (MMPs) [14].

Alcohol dehydrogenase, iron containing, 1 (*ADHFE1*) located on chromosome 8q13.1 was cloned by Deng and colleagues from a human fetal brain cDNA library [15]. *ADHFE1* is related to members of the group III metal-dependent alcohol dehydrogenase family [16], and encodes hydroxyacid-oxoacid transhydrogenase, which is responsible for the oxidation of 4-hydroxybutyrate to succinate semialdehyde [17]. The hypermethylation of *ADHFE1* was recently reported in CRC [18,19] and is associated with differentiation [20]. However, the association between the hypermethylation of *ADHFE1* and alcohol in CRC has not been reported yet.

In this study, the hypermethylation of *ADHFE1* was identified in CRC using quantitative methylation-specific polymerase chain reaction (QMSP). The expression level of *ADHFE1* in CRC tissues was compared to that in adjacent normal tissues using real-time reverse transcriptase-polymerase chain reaction (real-time PCR). We investigated the demethylating effects of *ADHFE1* using 5-aza-2′-deoxycytidine. We analyzed the effect of alcohol on methylation and expression of *ADHFE1* as well as cell proliferation in CRC cells.

## Methods

### Tissues

Fresh-frozen primary tumors (*n* = 73), paired tumors, and adjacent normal tissues (*n* = 73) from CRC patients were collected at the time of surgery at the Korea University Medical Center. The clinicopathologic features of CRC patients are summarized in Table 1. The “drinking group” comprised individuals consuming more than 300 mL of alcohol three or more times per a week and the “non-drinking” group contained individuals who did not consume alcohol. The tissues were collected after obtaining informed consent from the patients and the study was approved by the Institutional Review Board of Korea University (IRB No: KU-IRB-10-08-A-1). The diagnosis of CRC tissues was acquired from pathology reports histological evaluations.

**Table 1:** Clinicopathologic characteristics of colorectal cancer patients and methylation status of ADHFE1

**Characteristics**	**No. of cases**	**Methylation status of *ADHFE1 *** **(PMR, %)**	
		**Median (range)**	***p*-Value**
Normal	73	3.25 (±0.50)	< 0.001^†^
Colorectal cancer	73	74.89 (±8.06)	
Age (years)			0.957
≤ 65	35	75.35 (±13.73)	
> 65	38	74.46 (±9.13)	
Gender			0.114
Female	27	91.55 (±16.46)	
Male	46	65.10 (±8.21)	
Location			0.627
Colon	46	77.91 (±10.45)	
Rectum	27	69.73 (±12.77)	
TNM Stage			0.252
I,II	32	85.40 (±14.10)	
III,IV	41	66.68 (±9.18)	
Size (mm)			0.276
≤ 25	44	67.71 (±7.81)	
> 25	29	85.78 (±16.48)	
Alcohol consumption			0.012^†^
Non-drinking	36	95.27 (±13.15)	
Drinking	37	55.06 (±8.41)	

Statistical significance is evaluated by analysis of variance (ANOVA). ^†^*p-*Values of < 0.05 are considered statistically significant.

PMR: Percentage of methylated reference; TNM: Tumor, lymph nodes and metastasis.

### Cell lines

One normal colon cell line (CCD18Co) and 4 CRC cell lines (HT-29; SW480, Dukes’ type B; DLD-1, Dukes’ type C; LoVo, Dukes’ type C and stage IV) were obtained from the American Type Culture Collection (Manassas, VA, USA). CCD18Co cells were cultured in Eagle’s minimum essential medium and the 4 CRC cells were cultured in RPMI 1640 medium, all supplemented with 10% fetal bovine serum (Hyclone, Logan, UT, USA) and 1% penicillin/streptomycin (P/S; Life Technologies). The cells were maintained at 37°C and 5% CO_2_ atmosphere.

### Genomic DNA extraction

Genomic DNA was extracted using the QIAamp DNA Mini Kit (Qiagen, Valencia, CA, USA) according to the manufacturer’s recommendations. Tissue samples were ground up by 3-mm diameter punches and then mixed with 700 μL lysis buffer containing 20 μg/mL Labo Pass protease K (Cosmo Gene Tech., Seoul, Korea), 20 mM Tri∙HCl (pH 8.0), 5 mM EDTA (pH 8.0), 400 mM NaCl, and 1% SDS solution (Sigma-Aldrich, St. Louis, MO, USA). The mixed samples were incubated at 42°C overnight. After incubation, genomic DNA was purified by phenol/chloroform extraction, eluted in 100 μL of water, and quantified with a NanoDrop ND-100 device (Thermo Fisher Scientific, Hudson, NH, USA).

### Sodium bisulfite DNA modification

Two micrograms of genomic DNA in 20 μL of RNase-free water was bisulfite converted using the EpiTect fast DNA bisulfite kit (Qiagen) according to the manufacturer’s recommendations. The reaction was performed by mixing 85 μL of bisulfite mix solution and 35 μL of DNA protect buffer in 200 μL PCR tubes at room temperature. The bisulfite-converted genomic DNA was eluted from the column with 100 μL of dH_2_O and stored at −80°C until use.

### Quantitative methylation specific PCR (QMSP)

Quantitative methylation status of the bisulfite-converted genomic DNA was confirmed by quantitative real-time PCR using the 7500 Real-Time PCR System (Applied Biosystems, San Francisco, CA, USA) according to the manufacturer’s recommendations. Methylation primers were designed using the MethPrimer software (http://www.urogene.org/methprimer/). MSP primer sequences for the methylated sequence of *ADHFE1* (−100 to +202, position from translational start site +1): 5′- AGG GCG GTA TTT AAA TTT TTC GAA TT -3′ (sense), 5′- CGC GAA ACG AAT AAA CAA ACG CGA CCG A -3′ (antisense) ); reference sequence of beta-actin (*ACTB)* (−1645 to −1513): 5′- TGG TGA TGG AGG AGG TTT AGT AAG T −3′ (sense), 5′- AAC CAA TAA AAC CTA CTC CTC CCT TAA −3′ (antisense). The product sizes were 303 bp and 132 bp respectively. PCR reactions were performed using an optical 96-well tray in a final volume of 20 μL. The reaction mixture consisted of 5 μL of 2X Maxima SYBR Green/ROX qPCR master mix (Thermo Fisher Scientific), 250 nM of each primer, and 100 ng of bisulfite-converted DNA template. The QMSP program was as the following: 95°C for 10 min, followed by 45 cycles at 95°C for 15 s, and then 60°C for 1 min. After PCR, a thermal melt profile was performed to examine the homogeneity of the PCR application. Each DNA sample was analyzed in triplicate, and the mean quantity was used for further analysis. Relative quantification of the amplified gene levels in the bisulfite-converted genomic DNA sample was performed by measuring the threshold cycle (C_T_) values of *ADHFE1* and β-actin (*ACTB*). The mean quantity of genes was divided by the mean quantity of *ACTB* and was used for the normalization of input DNA. The negative values for *ACTB* were excluded from the methylation analysis. The bisulfite-converted genomic DNA of a known concentration was prepared at 1, 1/4, 1/16, and 1/64 by serial dilutions, and used in a standard curve for quantification. The modified genomic DNA by CpG methyltransferase *M.Sss*I (NEB, Ipswich, MA, USA) was used as a positive control according to the manufacturer’s recommendations. DNA methylation according to *M.Sss*I was verified using the restriction enzyme *Bst*UI (NEB).

### mRNA extraction and cDNA synthesis

mRNA was extracted using an RNeasy Mini kit (Qiagen) according to the manufacturer’s recommendations. mRNA was eluted in 20 μL of diethyl pyrocarbonate (DEPC) water (Qiagen) and quantified with a NanoDrop ND-100 device (Thermo Fisher Scientific). cDNA was synthesized from 1 μg of mRNA from each sample using Moloney murine leukemia virus reverse transcriptase (M-MLV RT) and random hexamers (Promega, Madison, WI, USA). The cDNA synthesis reaction was prepared according to the manufacturer’s recommendations by mixing 1 μg mRNA, 4 μL of 5× RT buffer, 1 μL of 500 nM oligo dT, 1 μL of a 10 mM dNTP solution, 0.5 μL of RNasin, 1 μL of M-MLV RT, and 12.5 μL of distilled water in PCR tubes. The mixture was incubated in 37°C for 1 h. cDNA was diluted with 20 μL of distilled water and stored at −80°C until use.

### Real-time PCR

mRNA expression was confirmed by quantitative real-time PCR using a 7500 Real-Time PCR System (Applied Biosystems) according to the manufacturer’s recommendations. The primers were designed using Primer3 version 0.4.0 (http://primer3.ut.ee/). The specific primers were: *ADHFE1*: 5′- TGC CAT TTT TGA CTA TGA ACA CTT -3′(sense), 5′- GAC AGC CCT CTT CAG ATA CTT AGC -3′(antisense); *ACTB*, 5′- AGA GCT ACG AGC TGC CTG AC −3′ (sense) and 5′- AGC ACT GTG TTG GCG TAC AG −3′ (antisense. The product sizes of *ADHFE1* and *ACTB* were 304 bp and 184 bp, respectively. The PCR reaction was performed in a final volume of 20 μL using an optical 96-well tray. The reaction mixture consisted of 5 μL of 2× Maxima® SYBR Green/ROX qPCR Master Mix (Thermo Fisher Scientific), 250 nM of each primer, and 100 ng of cDNA template. The real-time PCR program was initiated at 95°C for 10 min, followed by 35 cycles of 95°C for 15 s and 60°C for 1 min. The thermal melt profile was examined to assess the homogeneity of the PCR application. Each DNA sample was analyzed in triplicate, and the mean quantity was used for further analysis. The relative levels of amplified mRNA in each sample were quantified by measuring the threshold cycle (C_T_) values of target genes. The mean quantity of each gene was divided by the mean quantity of *ACTB* and was used for the normalization of input DNA. cDNA of a known concentration was prepared at 1, 1/10, 1/100, and 1/1000 by serial dilutions and used as the standard curve for quantification.

### Chemical treatment

To determine the optimal concentration of ethanol (Sigma-Aldrich) in a normal colon cell line and 4 CRC cell lines, we measured cell viability with the 3-(4,5-dimethylthiazol-2-yl)-2,5-diphenyltetrazolium (MTT) assay (data not shown) according to the manufacturer’s recommendations using MTT reagents (10 μL/well, 7.5 mg/mL in phosphate-buffered saline, PBS) and dimethyl sulfoxide (50 μL/well, Sigma-Aldrich). To identify the alteration of methylation status by treatment with ethanol, cells were seeded in 6-well culture plates (SPL LifeScience, Pocheon, Korea) at a density of 0.5 × 10^5^ cells per well. After 24 h, the cells were treated with 100 mM ethanol for 72 h at 37°C in a 5% CO_2_ atmosphere. The cells were washed in PBS three times and then harvested. The cells were seeded at a density of 0.5 × 10^5^ cells/well in a 6-well plate, and after 24 h, the cells were treated with demethylation agent 30 μM 5-aza-2′-deoxycytidine (5-aza-dC) for 72 h. The results were drawn from experiments repeated at least three times.

### ADHFE1 siRNA treatment

CCD18Co and DLD-1 cells were seeded at a density of 0.5 × 10^5^ cells/well in a 6-well plate and allowed to grow to 70% confluence for 24 h. Cells were serum-starved for 30 min before siRNA transfection. Transient transfections were performed using a transfection reagent (Lipofectamine 2000; Life Technologies, Carlsbad, CA, USA) according to the manufacturer’s protocol. A commercial *ADHFE1* siRNA was purchased from Qiagen. The specific primers were: *ADHFE1* siRNA: 5′- GGA UGU UGA UGA UGG CCU ATT -3′ (sense), 5′- UAG GCC AUC AUC AAC AUC CAG -3′ (anti-sense); non-target siRNA: 5′- UUC UCC GAA CGU GUC ACG UTT -3′ (sense), 5′- ACG UGA CAC GUU CGG AGA ATT -3′ (anti-sense). Fifty nM of siRNA and 6 μL of transfection reagent were each diluted first with 100 μL of reduced serum media, and then mixed. The mixtures were allowed to incubate for 10 minutes at room temperature and then added dropwise to each culture well containing 1 mL of reduced serum media. After 4 h, the medium was changed using fresh complete medium. Cells were cultivated for 24 h, 48 h, or 72 h. The cells were washed in PBS (Sigma-Aldrich) three times and then harvested.

### Cell counting assay and cell image capture

After treatment with indicated agents, CCD18Co and DLD-1 cells in 6-well plates were washed with PBS (Sigma-Aldrich). The cells were detached with 0.05% trypsin (Wellgene, Deagon, Korea) and 0.53 mM EDTA (Wellgene, Deagon, Korea) for 2 min at 37°C. Eight hundred microliters of media supplemented with 10% FBS (Hyclone) was then added, and 20 μL of cells were combined with 20 μL of 0.4% trypan blue solution (Sigma-Aldrich). The retained cells were transferred to a counting chamber (Paul Marienfeld GmbH & Co. KG, Lauda-Königshofen, Germany) and cells were counted using Nikon TMS inverted stage microscope (Nikon Instrument Inc., Melville, NY, USA). For counter staining, cells were fixed with 4% paraformaldehyde (Sigma-Aldrich) for 15 min at room temperature, and the fixed cells were washed three times in PBS (Sigma-Aldrich). Cells were stained with Hoechst 33342 (Sigma-Aldrich) for 10 min at room temperature without light and washed with PBS (Sigma-Aldrich). Images of cells were obtained using a LSM 700 Confocal Laser Scanning Microscope (Carl Zeiss Co., Ltd., Jena, Germany). The results were drawn from experiments repeated at least three times.

### Immunoblot analysis

Cells were grown in 6-well plates and treated with the indicated agents. Following treatment, the media was aspirated and the cells were washed three times in ice-cold PBS (Sigma-Aldrich) and lysed in 100 μL of lysis buffer. The samples were then briefly sonicated, centrifuged for 5 min, and supernatants were boiled for 5 min at 95°C. The supernatants were subjected to electrophoresis on sodium dodecyl sulfate polyacrylamide gel electrophoresis (10%) gels, and transferred to polyvinylidene difluoride membranes. The blots were incubated overnight at room temperature with primary *ADHFE1* antibody (Sigma-Aldrich), and then washed six times in Tris-buffered saline/0.1% Tween 20 prior to incubation with horseradish peroxidase-conjugated secondary antibody (anti-rabbit, Sigma-Aldrich) for 1 h at room temperature. The blots were visualized using ECL (Amersham Biosciences, Buckinghamshire, UK). Beta-actin (*ACTB*) and glyceraldehyde-3-phosphate dehydrogenase (*GAPDH*) were used as loading controls.

### Fluorescence-activated cell sorting (FACS) analysis

Cells treated with propidium iodide (PI) can be utilized not only for assessment of the stages of the cell cycle (G0/G1, S, G2/M), but also for identification of apoptotic cells (hypodiploid, sub G0 peak). Half a million cells were pelleted at 1000 × g for 5 min and were mixed with 10 μl of Vindelov’s PI (Sigma-Aldrich) stain solution (1.21 g TRIS base, 584 mg NaCl, 10 mg RNAse, 50.1 mg PI, 1 ml Nonidet P40, pH 8.0) and 490 μl of standard azide buffer(PBS buffer, 0.5% EDTA pH 8.0). Cells were incubated at 4°C overnight and analyzed using an FACS caliber flow cytometer (Becton-Dickinson, NJ, USA) at an excitation wavelength of 488 nm and an emission wavelength of 525 nm. The 10,000 cells were measured by gating the polymorphonuclear leukocytes (PMN) population and analyzed using the BD FACSDiva Software version 6. 1. 3 (Becton-Dickinson).

### Statistical analysis

MTT assay results were quantified using SoftMax® Pro software (Molecular Devices, LLC). The methylated intensity ratio of QMSP was determined as the percentage of methylated reference (PMR), and the PMR value was defined as: [(*ADHFE1*)_sample_/(*ACTB*)_sample_]/[( *ADHFE1*)_M.SssI_/(*ACTB*)_M.SssI_] × 100. A PMR value of ten or more indicated hypermethylation. The significance of the differences in PMR values was defined by the chi-squared test, Fisher’s exact test, and ANOVA using Sigma Stat (SPSS Inc., Chicago, IL, USA). In all statistical tests, *p*-Values of *<* 0.05 were considered statistically significant. Real-time PCR and immunoblot analysis data were compared and qualitative differences between samples were analyzed using Sigma Stat (SPSS Inc.). *p*-Values of < 0.05 were considered statistically significant.

## Results

### *ADHFE1* is hypermethylated and down regulated in CRC

**Figure 1:**
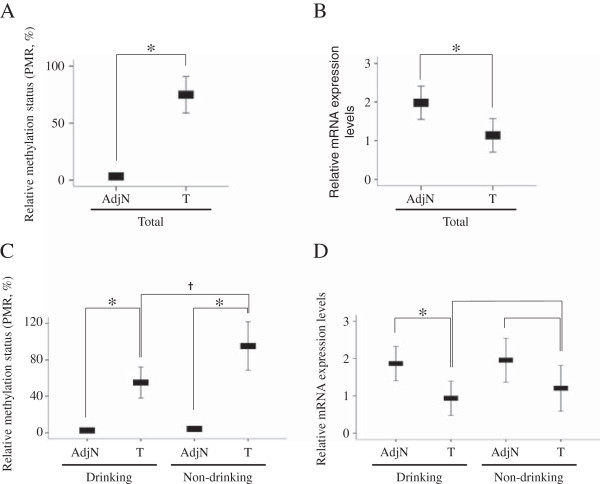
**The methylation status and mRNA expression levels of *ADHFE1 *in CRC tissues and adjacent normal tissues.** The methylation status of *ADHFE1* in 73 CRC tissues and adjacent normal tissues is assessed using QMSP. **A**, **C**. *ADHFE1* is hypermethylated in CRC **(A)** and also significantly hypermethylated in drinking groups and non-drinking groups **(C)**. **B**. The expression levels of *ADHFE1* in 73 CRC tissues compared with adjacent normal tissues was determined in 73 CRC tissues by real-time PCR. The mRNA expression of *ADHFE1* is significantly decreased in CRC compared to adjacent normal tissues. **D**. The down regulation of *ADHFE1* is presented in drinking groups and non-drinking groups. *,†*p-*Values of < 0.05 were considered statistically significant. AdjN: Adjacent normal tissue; T: Colorectal cancer tissues; PMR,: Percentage of methylated reference.

To determine the methylation status of *ADHFE1*, we analyzed the methylation status of 73 CRC tissues and adjacent normal tissues using QMSP. *ADHFE1* was hypermethylated in 69 out of 73 CRC tissues (95%) and only 2 out of 73 adjacent normal tissues (3%). Comparative analysis using percentage of methylated reference (PMR) value also indicated that the methylation status of *ADHFE1* was much higher in CRC tissues compared to adjacent normal tissues (Figure 1A). To verify whether the mRNA expression of *ADHFE1* is caused by aberrant DNA methylation, we examined the expression level of *ADHFE1* in 73 CRC tissues and adjacent normal tissues by real-time PCR. The mRNA expression of *ADHFE1* was significantly reduced in CRC tissues compared to adjacent normal tissues (Figure 1B). We performed statistical analysis between the methylation of *ADHFE1* and clinicopathologic features of CRC. The PMR value of *ADHFE1* was not significantly different in most clinicopathologic features except the alcohol consumption in CRC tissues (Table 1). The methylation status of *ADHFE1* in CRC tissues was much higher in the non-drinking group than the drinking group (*p* < 0.05, Figure 1C). However, mRNA expression of *ADHFE1* in CRC tissues, compared to adjacent normal tissues, was more reduced in groups of drinking and old age (Figure 1D, Table 2).

**Table 2:** **mRNA expression of *ADHFE1 *** **associated with CRC risk factors**

**Characteristics**	**mRNA expression of *ADHFE1***		***p*-value**
	**AdjN**	**T**	
		**Median (range)**		**Median (range)**
Colorectal cancer	1.98 (±0.21)	1.14 (±0.22)	0.007^†^
Age (years)			
≤ 65	1.97 (±0.52)	1.54 (±0.41)	0.525
> 65	1.94 (±0.29)	1.04 (±0.30)	0.034^†^
Alcohol consumption			
Non-drinking	1.96 (±0.29)	1.21 (±0.30)	0.078
Drinking	1.87 (±0.23)	0.94 (±0.23)	0.005^†^

Statistical significance is evaluated by analysis of variance (ANOVA). ^†^*p-*Values of < 0.05 are considered statistically significant.

AdjN: Adjacent normal tissue; T: Colorectal cancer tissues.

### Restoration of *ADHFE1* expression by 5-aza-dC in CRC cells

**Figure 2:**
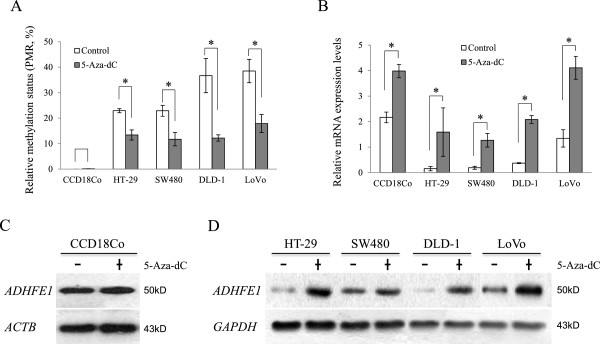
**Changes of *ADHFE1 *methylation and expression by treatment with 5-aza-dC in CRC cells and normal colon cells.** After treatment with 5-aza-dC in cells, the methylation status and expression levels of *ADHFE1* are observed using QMSP, real-time PCR and immuno-blotting analysis. **A**. *ADHFE1* is hypermethylated in 4 CRC cells compared to normal colon cells and significantly demethylated in 4 CRC cells by 5-aza-dC. **B**. The mRNA expression of *ADHFE1* is reduced in 4 CRC cells but increased in all cells treated with 5-aza-dC. **C**. The protein expression of *ADHFE1* is not affected by 5-aza-dC in CCD18Co. **D**. Protein expression of *ADHFE1* is restored by 5-aza-dC in 4 CRC cells. Expression of *ACTB* and *GAPDH* is used as a loading control. *p-Values of < 0.05 were considered statistically significant. +: Treated with 5-aza-dC; −: Non-treated with 5-aza-dC; PMR: Percentage of methylated reference.

The effect of methylation on mRNA expression was determined by MSP and real-time PCR analysis in 5-aza-dC treated HT-29, SW-480, DLD-1, LoVo, and CCD18Co cells. *ADHFE1* was hypermethylated in 4 CRC cells and demethylated by treatment with 5-aza-dC (Figure 2A). The mRNA expression of *ADHFE1* was relatively reduced in 4 CRC cells compared with CCD18Co and was restored by treatment with 5-aza-dC (Figure 2B). To confirm the protein expression of *ADHFE1*, we measured *ADHFE1* protein levels and estimated the change in protein expression by 5-aza-dC in all cell lines using western blot analysis. The basal protein level of *ADHFE1* was lower in 4 CRC cells compare to that of CCD18Co cells, and protein expression of *ADHFE1* in 4 CRC cells increased upon treatment with 5-aza-dC (Figure 2C). These results suggest that promoter methylation of *ADHFE1* regulates the expression of *ADHFE1.*

### Alcohol down-regulates the expression of *ADHFE1* by methylation in CRC cells

**Figure 3:**
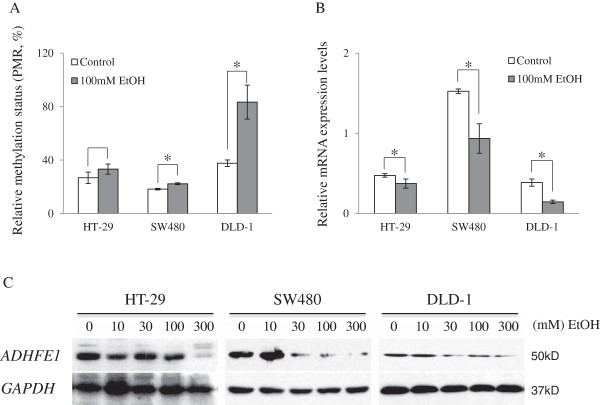
**Changes in methylation and expression of *ADHFE1 *by ethanol treatment.** The methylation and expression changes of *ADHFE1* are determined in HT-29, SW480, and DLD-1 cells by treatments with ethanol using QMSP, real-time PCR and immunoblot analysis. **A**. The methylation status of *ADHFE1* is significantly increased by ethanol treatment in 2 CRC cells. **B**. *ADHFE1* expression is diminished by ethanol in 3 CRC cells. **C**. After treatment with ethanol in CRC cells, the protein expression of *ADHFE1* is decreased in a concentration-dependent manner. Expression of *GAPDH* is used as a loading control. *p-Values of < 0.05 were considered statistically significant; PMR: Percentage of methylated reference.

To examine the correlation between *ADHFE1* methylation and expression in the presence and absence of alcohol, the methylation status and mRNA levels of *ADHFE1* were assessed by QMSP and real-time PCR analysis, respectively, in 3 CRC cells after ethanol treatment. The methylation status of *ADHFE1* was significantly induced in SW480 and DLD-1 cell (Figure 3A), whereas its expression was decreased in HT-29, SW480, and DLD-1 cells by treatment with 100 mM ethanol for 3 days (Figure 3B). In addition, we investigated the protein levels of *ADHFE1* in 3 CRC cells after treatment with various concentrations of ethanol for 3 days. After treatment with ethanol, the protein expression of *ADHFE1* was decreased in a concentration-dependent manner in both cells (Figure 3C). These results suggest that alcohol promotes the hypermethylation of *ADHFE1* and methylation-mediated silencing of *ADHFE1*.

### Alcohol induces the proliferation of CRC cells and down-expression of *ADHFE1*

**Figure 4:**
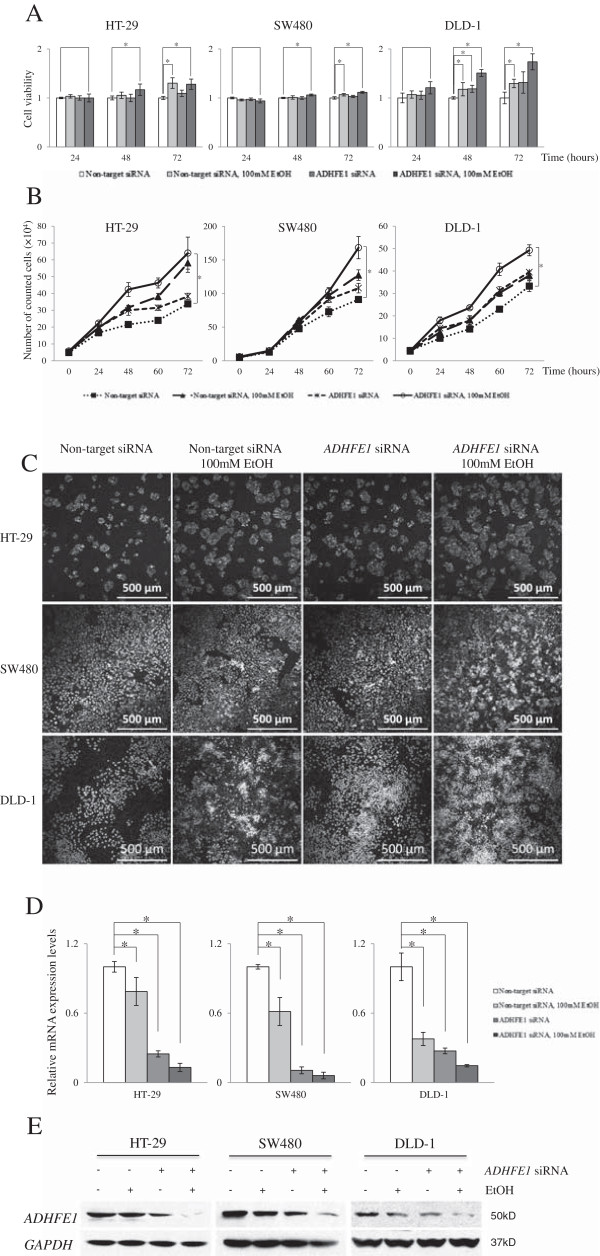
**The effect of *ADHFE1 *down regulation on cell viability and proliferation.** The cell viability and proliferation of HT-29, SW480, and DLD-1 cells after ethanol treatment, transfection of *ADHFE1* siRNA and combined treatment are determined by MTT, cell counting, and counter staining assay. **A**. Cell viability of 3 CRC cells is significantly increased by treatment with ethanol, siRNA, and combination of both. **B**. Cell proliferation of 3 CRC cells is significantly increased by ethanol, siRNA, and co-treatment. **C**. The captured images of cells using Hoechst 33342 show that the number of 3 CRC cells is increased by ethanol, siRNA, and co-treatment. **D**. The mRNA expression of *ADHFE1* is significantly decreased in 3 CRC cells by treatment with ethanol, siRNA, and combination of both. **E**. *ADHFE1* protein expression is decreased in 3 CRC cells by treatment with ethanol, siRNA, and combination of both. *GAPDH* was used as a loading control. *p-Values of < 0.05 were considered statistically significant. +: Treated with agent; −: Non-treated with agent.

To investigate the function of *ADHFE1*, its expression was inhibited by treatment with ethanol and transfection of *ADHFE1* siRNA in HT-29, SW480, and DLD-1 cells. Cell viability and cell proliferation were then analyzed using MTT and a cell counting assay. The cell viability of HT-29, SW480, and DLD-1 cells was significantly increased by treatment with a combination of ethanol and *ADHFE1* siRNA. HT-29 cells was increased the cell viability by ethanol as much as co-treatment (Figure 4A).The cell counting assay showed that proliferation of 3 CRC cells was increased by ethanol and siRNA. Growth of DLD-1 cells by co-treatment was significantly higher than each treatment with alcohol and siRNA (Figure 4B). To confirm the effect of *ADHFE1* down regulation on cell proliferation, cells were stained with Hoechst 33342 after ethanol treatment and siRNA transfection. The number of HT-29, SW480, and DLD-1 cells was increased by ethanol, siRNA, and co-treatment (Figure 4C). Furthermore, mRNA and protein expression of *ADHFE1* were significantly decreased in 3 CRC cells by treatment with alcohol, siRNA, and co-treatment (Figure 4D, 4E). These results suggest that methylation-mediated down regulation of *ADHFE1* by alcohol may be associated with cell proliferation of CRC cells.

## Discussion

Recently, aberrant methylation of *ADHFE1* promoter was identified by a genome-wide methylation profiling screen using an array-based chip assay [18,19], and hypermethylation was found to be associated with CRC differentiation [20]. In this study, we also identified the hypermethylation of *ADHFE1* in CRC tissues compared to adjacent normal tissues using QMSP analysis. The PMR value of methylation status by QMSP was dichotomized at 4 PMR or 10 PMR for statistical purposes, as described previously [21,22]. In our experiments, 10 PMR or higher was considered as a cutoff to indicate a methylated state, whereas less than 10 PMR was considered as a cutoff to indicate an unmethylated state. Interestingly, the methylation status of *ADHFE1* in CRC tissues was higher in the non-drinking group than in the drinking group, but the expression of *ADHFE1* in CRC tissues compared to adjacent normal tissues was lower in the drinking group (Figure 1). The inverse correlation between *ADHFE1* methylation and expression was highly significant in the drinking group. Based on this result, promoter methylation of *ADHFE1* may be the main factor affecting its expression in the drinking group.

The age-associated hypermethylation and methylation-mediated silencing of several genes was reported in human prostate [23], colon tissues [24] and peripheral blood [25]. Based on the results presented here, we found no age-dependent *ADHFE1* methylation but we identified that *ADHFE1* down regulation in CRC tissues compared to adjacent normal tissues was more significant in the old age group than the young age group. The inverse correlation between methylation and expression of *ADHFE1* in 9 out of 12 CRC cell lines by 5-aza-dC was reported by Tae C.H. et al. [20]. In this study, we obtained the same results in 4 CRC cell lines (Figure 2). This result suggests that the promoter methylation of *ADHFE1* may be the key regulator of *ADHFE1* expression in CRC cells.

Alcohol is a major risk factor well known for the progression of CRC [10,26] and alters the methylation status of genes. Hypermethylation of alpha synuclein was significantly presented in peripheral blood of patients with chronic alcoholism [27]. Recently, array-based study has reported that various genes were hypermethylated in peripheral blood of patients with alcohol dependency compared to healthy controls [28]. We found that alcohol induced the hypermethylation of *ADHFE1* and decreased its expression in CRC cells as well as normal colon cells. Down regulation of *ADHFE1* by siRNA affected cell growth in CRC cells. The survival rate and cell growth of SW480 and DLD-1 cells were significantly increased by treatment with a combination of ethanol and *ADHFE1* siRNA than each individual treatment (Figure 4). The result demonstrates a synergetic effect between ethanol and *ADHFE1* siRNA. The viability of HT-29 cells was affected more by the treatment with ethanol than down expression of *ADHFE*. In addition, we confirmed the viability of CRC cells after co-treatment with 5-aza-dC and ethanol. The viability of CRC cells was significantly decreased after treatment with 5-aza-dC. On the other hand, co-treated cells had smaller range of decline compared with 5-aza-dC treated cells in HT-29 and SW480. The viability of DLD-1 cells were not affected by co-treatment (Additional file 1: Figure S1). In contrast, the down regulation of *ADHFE1* by ethanol and siRNA had opposite effects on the viability and proliferation of CCD18Co, normal colon fibroblast cells (Additional file 2: Figure S2). CCD18Co was increased rate of apoptosis by treatment with ethanol, siRNA, and combination of both, but DLD-1 was not affected (Additional file 3: Figure S3). Previous studies have found that several genes, including interleukin 6 and tumor necrosis factor-alpha, have opposite functions in normal and tumor cells [29,30]. Taken together, our results provide evidence for different functions of *ADHFE1* in normal colon cells and CRC cells.

## Conclusions

We found that *ADHFE1* was hypermethylated in CRC tissues compared to adjacent normal tissues and that the expression of *ADHFE1* was significantly reduced in the alcohol drinking and old age group. In addition, alcohol induced the hypermethylation of *ADHFE1* and decreased its expression in HT-29, SW480, and DLD-1 cells. The down regulation of *ADHFE1* by alcohol and *ADHFE1* siRNA induced the growth of 3 CRC cells, and co-treatment with alcohol and siRNA greatly increased cell proliferation in SW480 and DLD-1 cells. These results suggest that hypermethylation of the *ADHFE1* promoter by alcohol leads to a decrease in *ADHFE1* expression and methylation-mediated silencing of *ADHFE1* may be induced the progression of CRC cells.

## Abbreviations

ACTB: Beta-actin; ADH: Alcohol dehydrogenase; ADHFE1: Alcohol dehydrogenase, iron containing, 1; CRC: Colorectal cancer; CYP2E1: Cytochrome P450 subenzyme 2E1; EGFR: Epidermal growth factor receptor; EMT: Epithelial-mesenchymal transition; MMPs: Matrix metalloproteinases; FACS: Fluorescence-activated cell sorting; GAPDH: glyceraldehyde-3-phosphate dehydrogenase; MTT: 3-(4,5-dimethylthiazol-2-yl)-2,5-diphenyltetrazolium; QMSP: Quantitative methylation specific polymerase chain reaction; PBS: Phosphate-buffered saline; Real-time PCR: Real-time reverse transcription polymerase chain reaction; ROS: Reactive oxygen species; SAM: S-adenosylmethionine; TNM: Tumor, lymph nodes and metastasis; 5-aza-dC: 5-aza-2′-deoxycytidine.

## Competing interests

The authors declare to have no competing interests.

## Authors’ contributions

JWM designed the study and drafted the manuscript. SKL and YWL prepared the clinical specimens and participated in the organization of clinical data. JOL, NK, and HJL participated in the analysis of *in vitro* analysis. JSS helped with the organization of the results. JK provided the clinical specimens and clinicopathologic information. HSK helped with the analysis of the results and revised the manuscript. SHP supervised the study and revised the manuscript. All authors have read and approved the final manuscript.

## Pre-publication history

The pre-publication history for this paper can be accessed here:

http://www.biomedcentral.com/1471-2407/14/377/prepub

## Supplementary Material

Additional file 1: Figure S1 The effect of ethanol, 5-aza-dC and co-treatment on the viability of CRC cells. The viability of HT-29, SW480, and DLD-1 cells after treatment ethanol, 5-aza-dC, and combination of both is determined by MTT assay. The viability of HT-29, SW480, and DLD-1 cells is reduced by treatment with 5-aza-dC. However, viability of HT-29 and SW480 cells treated with both 5-aza-dC and ethanol has smaller range of decline, compared to those treated with 5-aza-dC. The viability of DLD-1 cells is not affected by co-treatment with 5-aza-dC and ethanol. *Indicates the increase in cell viability by treatment with agent. †Indicates the decrease in cell viability by treatment with agent. Click here for file

Additional file 2: Figure S2 The effect of *ADHFE1* down regulation on cell viability and proliferation in normal colon cells. The cell viability and proliferation of CCD18Co after ethanol treatment, transfection of *ADHFE1* siRNA, and combined treatment are determined by MTT, cell counting, and counter staining assay. A. The viability of CCD18Co cells is significantly decreased by ethanol, siRNA, and co-treatment. B. The proliferation of CCD18Co cells is significantly decreased by ethanol, siRNA, and co-treatment. C. The captured images of CCD18Co using Hoechst 33342 show that the number of CCD18Co cells is decreased by ethanol, siRNA, and co-treatment. D. *ADHFE1* protein expression is decreased in CCD18Co cells treated with ethanol, siRNA, and combination of both. *GAPDH* was used as a loading control. *p-Values of < 0.05 were considered as statistically significant. +: Treated with agent; −: Treated without agent. Click here for file

Additional file 3: Figure S3 The effect of *ADHFE1* down regulation on apoptosis in CCD18Co and DLD-1 cells. Apoptosis of CCD18Co and DLD-1 after ethanol treatment, transfection of *ADHFE1* siRNA, and combined treatment is determined by FACS analysis. Apotosis of CCD18Co cells is induced by ethanol, *ADHFE1* siRNA, and co-treatment, but that of DLD-1 cell are not affected. Click here for file
