# Supplementary material for: Alcohol induces cell proliferation via hypermethylation of ADHFE1 in colorectal cancer cells
Source: BMC Cancer. 2014 May 28;14:377. doi: 10.1186/1471-2407-14-377 (PMC4057807; doi:10.1186/1471-2407-14-377)
Supplement: Additional file 1: Figure S1 — The effect of ethanol, 5-aza-dC and co-treatment on the viability of CRC cells. The viability of HT-29, SW480, and DLD-1 cells after treatment ethanol, 5-aza-dC, and combination of both is determined by MTT assay. The viability of HT-29, SW480, and DLD-1 cells is reduced by treatment with 5-aza-dC. However, viability of HT-29 and SW480 cells treated with both 5-aza-dC and ethanol has smaller range of decline, compared to those treated with 5-aza-dC. The viability of DLD-1 cells is not affected by co-treatment with 5-aza-dC and ethanol. *Indicates the increase in cell viability by treatment with agent. †Indicates the decrease in cell viability by treatment with agent. [file 1471-2407-14-377-S1.pptx]

## Slide 1
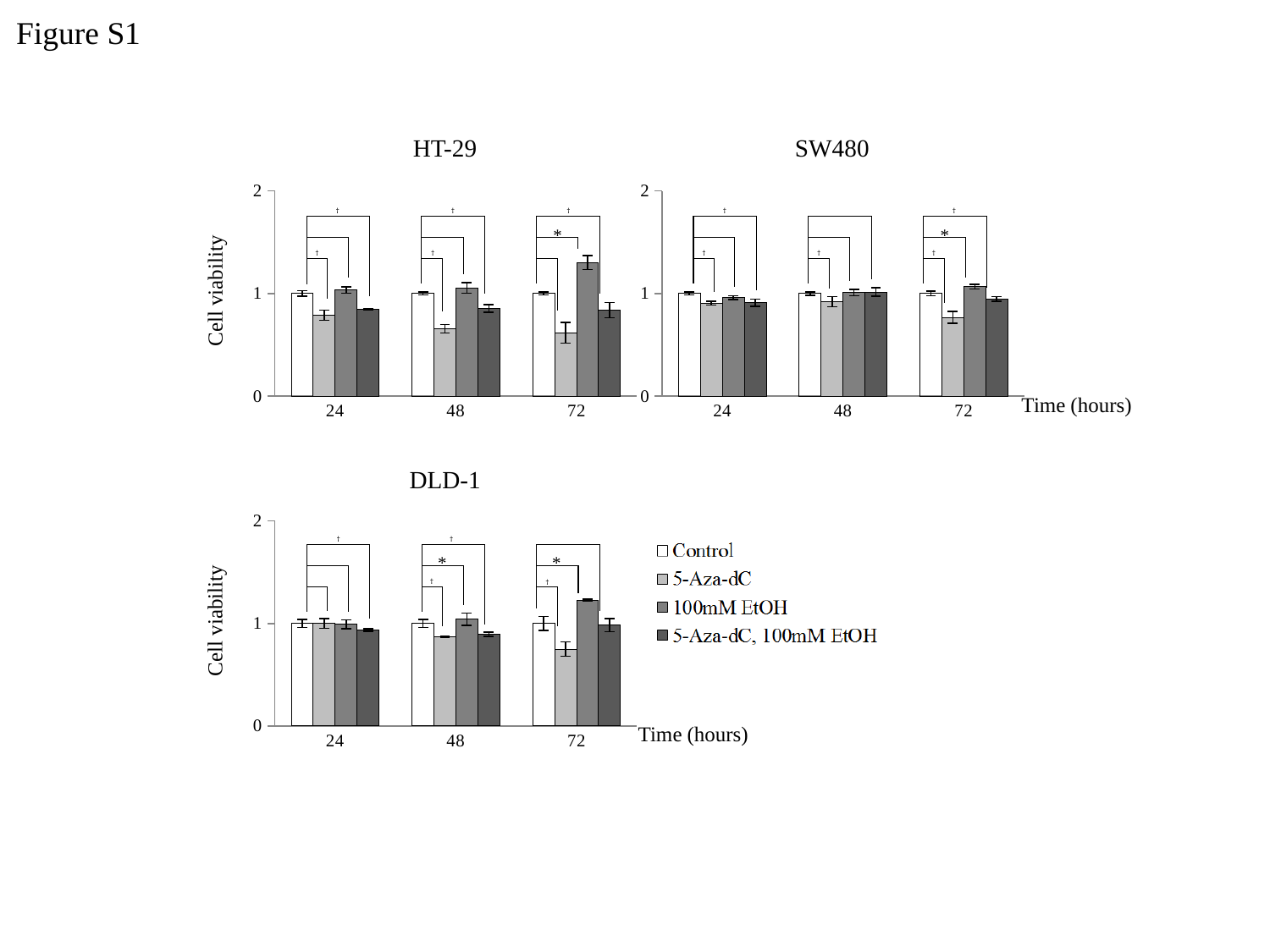

Figure S1
HT-29
SW480
### Chart
| Category | Control | 5-Aza-dC | 100mM EtOH | 5-Aza-dC, 100mM EtOH |
|---|---|---|---|---|
| 24 | 1.0 | 0.7876014146037029 | 1.0332737522342912 | 0.846765134179322 |
| 48 | 1.0 | 0.6570299500831946 | 1.0543412264723742 | 0.854747296173045 |
| 72 | 1.0 | 0.6168744360148392 | 1.3029197080291968 | 0.8374051669396078 |
### Chart
| Category | Control | 5-Aza-dC | 100mM EtOH | 5-Aza-dC, 100mM EtOH |
|---|---|---|---|---|
| 24 | 1.0 | 0.9057455825478797 | 0.9595796847635725 | 0.9090109075750908 |
| 48 | 1.0 | 0.9199916238462653 | 1.010937416635544 | 1.0145616211079074 |
| 72 | 1.0 | 0.7674207338795868 | 1.0666842924708442 | 0.9482543640897756 |†
†
†
†
†
*
*
†
†
†
†
†
Cell viability
Time (hours)
DLD-1
### Chart
| Category | Control | 5-Aza-dC | 100mM EtOH | 5-Aza-dC, 100mM EtOH |
|---|---|---|---|---|
| 24 | 1.0 | 0.9993353135450638 | 0.9927474918409284 | 0.9347776416093719 |
| 48 | 1.0 | 0.8727700739323943 | 1.0402731165374377 | 0.8950969213226909 |
| 72 | 1.0 | 0.7482611052795497 | 1.225976344565945 | 0.9829859388877996 |†
†
*
*
†
†
Cell viability
Time (hours)
