# Supplementary material for: Alcohol induces cell proliferation via hypermethylation of ADHFE1 in colorectal cancer cells
Source: BMC Cancer. 2014 May 28;14:377. doi: 10.1186/1471-2407-14-377 (PMC4057807; doi:10.1186/1471-2407-14-377)
Supplement: Additional file 2: Figure S2 — The effect of ADHFE1 down regulation on cell viability and proliferation in normal colon cells. The cell viability and proliferation of CCD18Co after ethanol treatment, transfection of ADHFE1 siRNA, and combined treatment are determined by MTT, cell counting, and counter staining assay. A. The viability of CCD18Co cells is significantly decreased by ethanol, siRNA, and co-treatment. B. The proliferation of CCD18Co cells is significantly decreased by ethanol, siRNA, and co-treatment. C. The captured images of CCD18Co using Hoechst 33342 show that the number of CCD18Co cells is decreased by ethanol, siRNA, and co-treatment. D. ADHFE1 protein expression is decreased in CCD18Co cells treated with ethanol, siRNA, and combination of both. GAPDH was used as a loading control. *p-Values of < 0.05 were considered as statistically significant. +: Treated with agent; −: Treated without agent. [file 1471-2407-14-377-S2.pptx]

## Slide 1
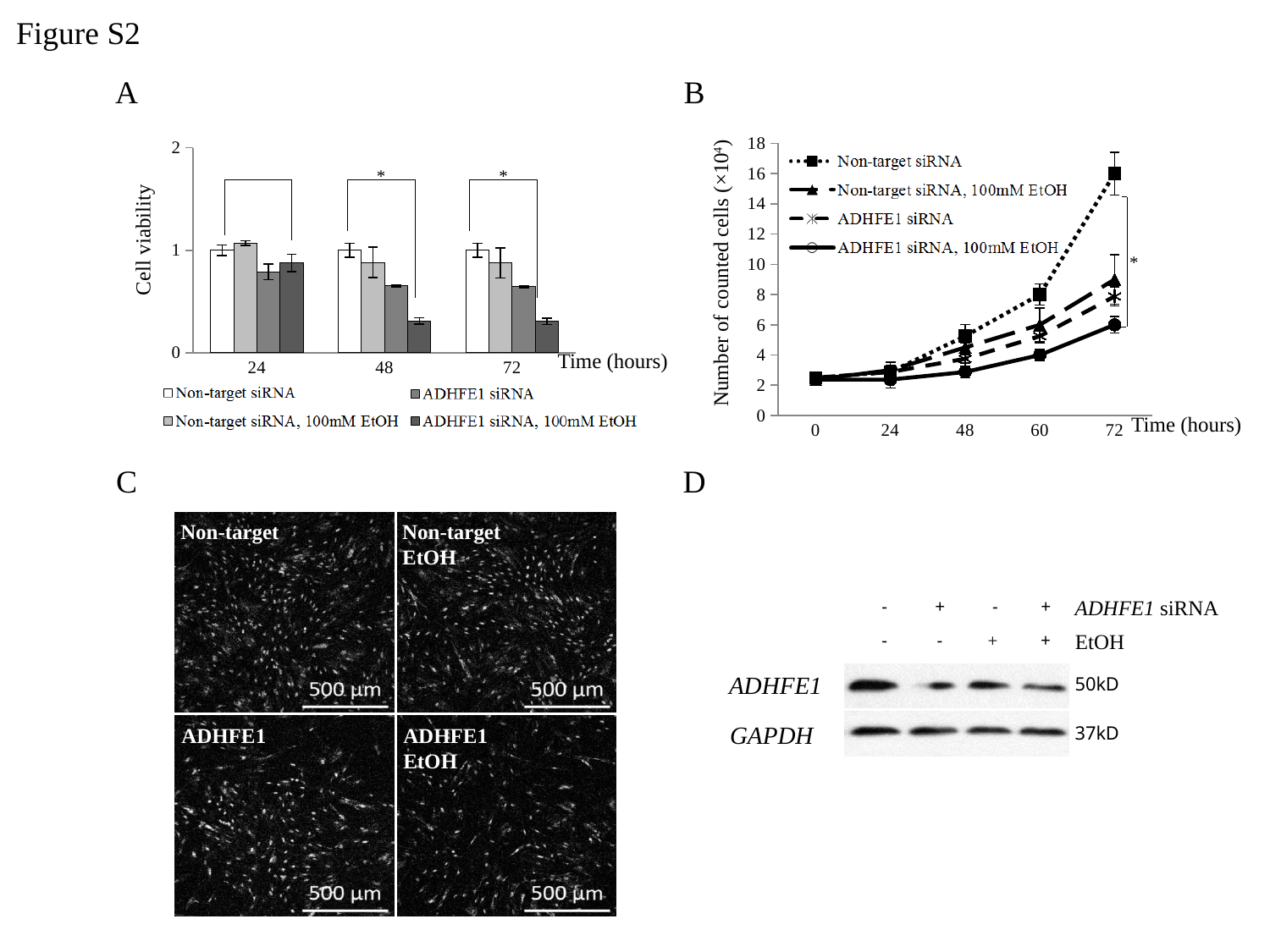

Figure S2
A
B
### Chart
| Category | | | | |
|---|---|---|---|---|
| 0 | 2.5 | 2.375 | 2.5 | 2.375 |
| 24 | 2.875 | 3.0 | 2.875 | 2.375 |
| 48 | 5.25 | 4.5 | 3.75 | 2.875 |
| 60 | 8.0 | 6.0 | 5.25 | 4.0 |
| 72 | 16.0 | 9.0 | 7.875 | 6.0 |*
Number of counted cells (×104)
Time (hours)
### Chart
| Category | Non-target siRNA | Non-target siRNA, 100mM EtOH | ADHFE1 siRNA | ADHFE1 siRNA, 100mM EtOH |
|---|---|---|---|---|
| 24 | 1.0 | 1.0682196339434278 | 0.7886855241264558 | 0.875207986688852 |
| 48 | 1.0 | 0.8806047966631908 | 0.6527632950990616 | 0.30969760166840465 |
| 72 | 1.0 | 0.8767051416579223 | 0.6442812172088145 | 0.30640083945435465 |*
*
Cell viability
Time (hours)
C
D
Non-target
Non-target
EtOH
ADHFE1
ADHFE1
EtOH
ADHFE1 siRNA
-
+
-
+
EtOH
-
-
+
+
ADHFE1
50kD
GAPDH
37kD
