# Supplementary material for: Alcohol induces cell proliferation via hypermethylation of ADHFE1 in colorectal cancer cells
Source: BMC Cancer. 2014 May 28;14:377. doi: 10.1186/1471-2407-14-377 (PMC4057807; doi:10.1186/1471-2407-14-377)
Supplement: Additional file 3: Figure S3 — The effect of ADHFE1 down regulation on apoptosis in CCD18Co and DLD-1 cells. Apoptosis of CCD18Co and DLD-1 after ethanol treatment, transfection of ADHFE1 siRNA, and combined treatment is determined by FACS analysis. Apotosis of CCD18Co cells is induced by ethanol, ADHFE1 siRNA, and co-treatment, but that of DLD-1 cell are not affected. [file 1471-2407-14-377-S3.pptx]

## Slide 1
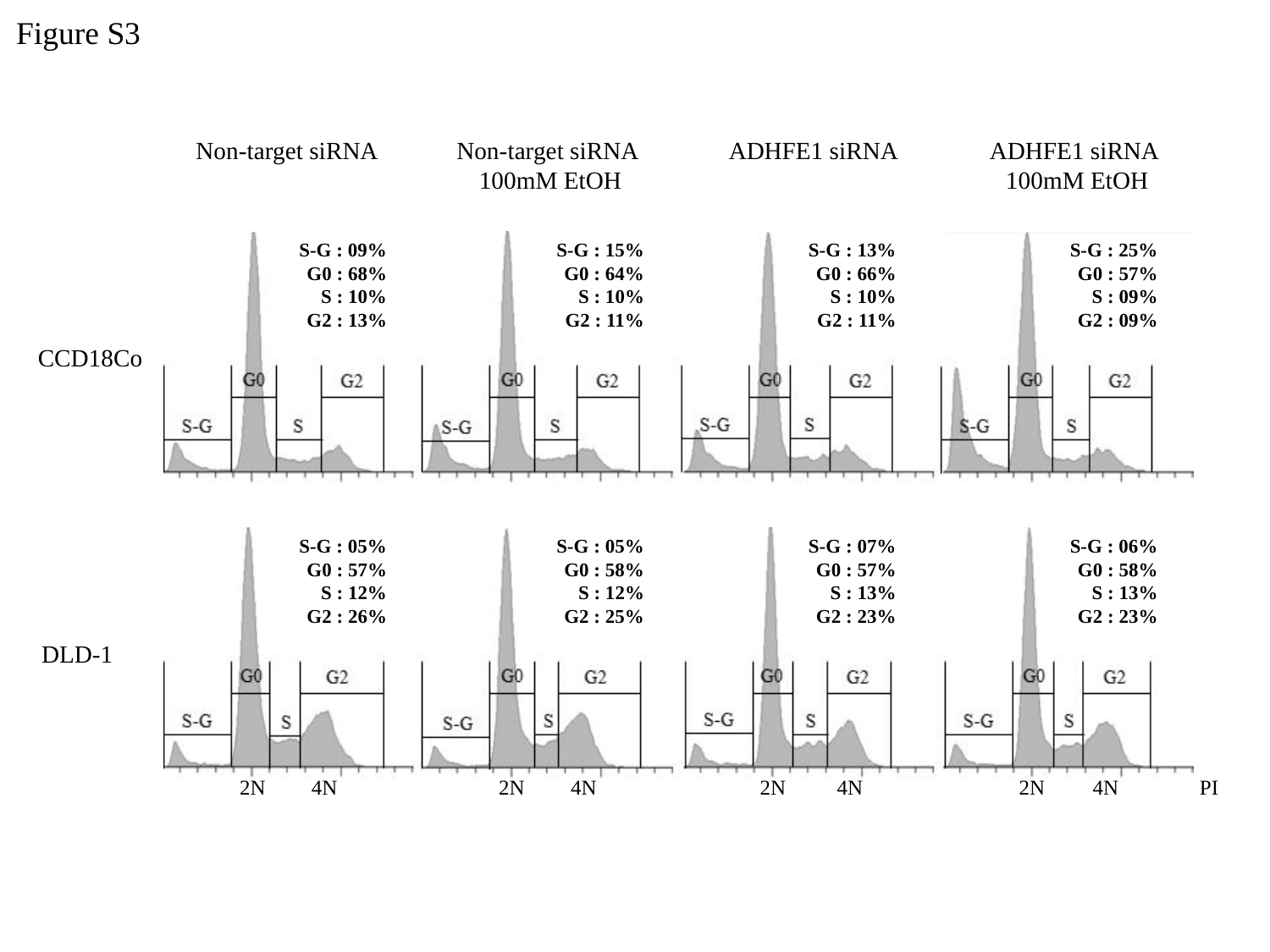

Figure S3
Non-target siRNA
Non-target siRNA
100mM EtOH
ADHFE1 siRNA
ADHFE1 siRNA
100mM EtOH
S-G : 09%
G0 : 68%
S : 10%
G2 : 13%
S-G : 15%
G0 : 64%
S : 10%
G2 : 11%
S-G : 13%
G0 : 66%
S : 10%
G2 : 11%
S-G : 25%
G0 : 57%
S : 09%
G2 : 09%
CCD18Co
S-G : 05%
G0 : 57%
S : 12%
G2 : 26%
S-G : 05%
G0 : 58%
S : 12%
G2 : 25%
S-G : 07%
G0 : 57%
S : 13%
G2 : 23%
S-G : 06%
G0 : 58%
S : 13%
G2 : 23%
DLD-1
2N
4N
2N
4N
2N
4N
2N
4N
PI
